# Maintenance of Islet Morphology Is Beneficial for Transplantation Outcome in Diabetic Mice

**DOI:** 10.1371/journal.pone.0057844

**Published:** 2013-02-25

**Authors:** Chloe L. Rackham, Peter M. Jones, Aileen J. F. King

**Affiliations:** Diabetes Research Group, Division of Diabetes and Nutritional Sciences, School of Medicine, King's College London, London, United Kingdom; University of Santiago de Compostela School of Medicine – CIMUS, Spain

## Abstract

We have previously shown that co-transplantation of islets and Mesenchymal Stem Cells (MSCs) improves islet graft function and revascularisation, which was associated with the maintenance of normal islet morphology. The aim of the current study was to determine whether maintaining islet morphology in the absence of additional islet-helper cells would improve transplantation outcome in diabetic mice. Islets were isolated from C57BL/6 mice. Recipient streptozotocin-diabetic C57BL/6 mice were transplanted with a minimal mass of 150 islets as a single pellet or islets that were either manually dispersed or dispersed within a matrigel plug beneath the kidney capsule. Blood glucose concentrations were monitored for one month. Islet graft morphology and vascularisation were analysed by histology. Islets dispersed either alone or within matrigel plugs maintained near normal morphology, in contrast to pelleted islets, where individual islets fused to form large endocrine aggregates. The vascularisation of manually dispersed islets and islets dispersed within matrigel plugs was increased relative to respective control pelleted islet grafts. After one month 1/6 mice transplanted with pelleted islets cured compared to 5/6 mice transplanted with manually dispersed islets. The curative capacity of islets dispersed in matrigel was also better than that of pelleted islets (5/8 islet-matrigel implanted mice vs. 1/7 mice transplanted with pelleted islets cured by one month). Therefore, this study demonstrates that the maintenance of islet morphology is associated with improved graft function and revascularisation in diabetic mice.

## Introduction

Allogeneic islet transplantation represents a viable therapy for the treatment of type 1 diabetes (T1D) in a selected group of patients. Remarkable improvements in the clinical islet transplantation field have been made with the development of the Edmonton protocol [1] and subsequent improvements on the original protocol [2]. However, the extensive loss of islets during the post-transplantation period means that individual graft recipients require multiple donors, further limiting the clinical applicability of islet transplantation as a therapy for T1D. Experimental studies in animal models are therefore being directed towards understanding the reasons for post-transplantation islet failure and to developing strategies to enhance the survival, function and engraftment of transplanted islets.

Delivering islets via the clinically-relevant intraportal route is technically challenging in experimental studies using rodents and it complicates subsequent graft retrieval for post-transplantation analysis, so extrahepatic sites are often used. In addition, while infusing islets into the hepatic portal vein is relatively simple and non-invasive in humans, experimental evidence is emerging that this site places the grafts into a hostile microenvironment which may be responsible, at least in part, for the post-transplantation loss of islet function [3], so the use of alternative sites may have clinical benefits. However, transplantation of islets as pellets at extrahepatic sites results in the fusion of individual islets and formation of large endocrine aggregates [4]–[6], which may be deleterious to their function. In a recent study in which we co-transplanted mesenchymal stem cells (MSCs) with islets beneath the kidney capsule in diabetic mice, we noted profound alterations in graft morphology when compared to islet alone grafts, with the MSCs maintaining normal islet size and architecture at the subcapsular site [6]. This was associated with increased vascularisation of the transplanted islets and beneficial outcomes for graft function and glycemic control when compared to islet-alone grafts. MSCs may influence graft function through multiple mechanisms [7]–[15], so in the current study we have investigated whether maintenance of islet morphology *per se* influences islet transplantation outcomes, in the absence of MSCs or any alternative supportive cell type. Specifically, we have used two different non-cell based experimental strategies to maintain islet morphology in the renal subcapsular site and assessed the effects on islet function *in vivo* compared to conventional implantation of islet pellets.

## Materials and Methods

### Ethics Statement

All animal procedures were approved by our institution's Ethics Committee and carried out under license, in accordance with the UK Home Office Animals (Scientific Procedures) Act 1986 (Project licence: PPL no. 70/6770). All animals had free access to water and pelleted food throughout experiments. For all surgical procedures mice were anesthetised with isofluorane. Buprenorphine was administered at a dose of 30 µg/kg, as an analgesic and all efforts were made to minimise suffering.

### Experimental animals

Male C567Bl/6 mice (Charles River, Margate, UK) aged 8–12 weeks were used as donors and recipients. Mice were made diabetic by i.p. streptozotocin (STZ) injection (180 mg/kg; Sigma-Aldrich, Poole, UK) and those with a non-fasting blood glucose concentration of ≥20 mmol/l were used as recipients. Blood glucose concentrations were determined using a blood glucose meter and strips (Accu-Chek; Roche, Burgess Hill, UK).

### Islet isolation

Islets were isolated by collagenase digestion (1 mg/ml; type XI; Sigma-Aldrich) followed by density gradient separation (Histopaque-1077; Sigma-Aldrich). After washing with RPMI-1640, islets were picked into groups of 150 for transplantation, as described previously [16].

### Transplantation of pelleted and manually dispersed islets

The first experimental series was designed to determine whether manually spreading islets out beneath the kidney capsule was able to maintain normal islet size and morphology. Mice were transplanted with 150 freshly isolated islets either as a single cluster of islet cells that had been centrifuged into pellets (pelleted islets transplant group) in PE50 polyethylene tubing (Becton Dickinson, Sparks, MD, USA) before placing underneath the kidney capsule using a Hamilton syringe (Fisher, Pittsburg, PA, USA). Alternatively, islets were suspended in media and aspirated into PE50 polyethylene tubing and sedimented by gravity. Islets were then spread out over the majority of the upper surface of the kidney capsule, using the Hamilton syringe (manually dispersed islet transplant group).

### Transplantation of islets dispersed in matrigel plugs

The second series of experiments used an alternative approach to maintain normal islet size and morphology, which was to disperse the islets in matrigel plugs beneath the kidney capsule. Matrigel (Becton Dickinson marathon growth factor reduced, high concentration) was kept at −20°C until use. 250 µl aliquots were defrosted at 4°C overnight before transplantation. Each aliquot was made up to 350 µl using PBS and 1 U heparin, before adding 150 freshly isolated islets whilst being careful to avoid any bubble formation. Matrigel has a liquid gelatinous state at 4°C, but solidifies at 37°C. Therefore, islet-matrigel preparations were kept on ice until immediately before transplantation. The matrigel solution was used to fill dead space in the Hamilton syringe and PE50 polyethylene tubing. 150 islets in the matrigel solution were aspirated into the tubing and then implanted beneath the kidney capsule, ensuring that the islet-matrigel solution was spread over the majority of the upper surface of the kidney.

### Graft function

The body weight and blood glucose concentrations of recipient mice were monitored every 3–4 days for a 1 month monitoring period. Cure was defined as non-fasting blood glucose concentrations ≤11.1 mmol/l for at least two consecutive readings, without reverting to hyperglycaemia on any subsequent day. At 1 month in some cured animals the graft-bearing kidney was removed to determine whether graft removal would result in reversion to hyperglycaemia. Mice were killed 3–4 days later and the graft-bearing kidney removed for histological analysis.

### Immunohistochemistry

Graft bearing kidneys and pancreata were fixed in 4% (vol./vol.) formalin and paraffin-embedded. Sections (5 µm thick) were stained for insulin, glucagon and microvascular endothelial cells (ECs). For CD34 staining (detection of ECs), antigen retrieval was required (2 min in 10 mmol/l citric acid solution pH 6.0 in a pressurised cooker). Sections were incubated for 1 h at room temperature in either polyclonal guinea pig anti-insulin antibody (1∶1000; Dako, Ely, UK) for the detection of β-cells, or with a monoclonal rat anti-CD34 antibody (1∶500 AbD serotec, Kidlington, UK) for the detection of ECs. Slides were then incubated for 1 h at room temperature with either a goat biotin anti-guinea pig antibody (1∶200; Jackson Immunolaboratories, West Grove, PA, USA) or a rabbit biotinylated anti-rat antibody (1∶200; Vector Laboratories, Peterborough, UK). Sections were counterstained with hematoxylin. For immunofluorescence labeling of insulin, a polyclonal guinea pig anti-insulin antibody (1∶100; Jackson) was used (1 h at room temperature) with a Texas Red anti-guinea pig secondary antibody (1∶40; Jackson; 1 h at room temperature). For immunofluorescence labeling of glucagon, a monoclonal mouse anti-glucagon antibody (1∶200; Sigma-Aldrich, Dorset, UK) was used (1 h at room temperature) with a FITC anti-mouse secondary antibody (1∶40; Jackson; 1 h at room temperature).

### Evaluation of graft morphology and vascular density

For each animal ≥5 tissue sections from different regions of the graft were analysed for vascular density. Graft morphology was evaluated by measuring the total endocrine area per graft section and extent of islet fusion as previously described [6]. Briefly, to evaluate the extent of fusion between individual islets, the area of individual endocrine aggregates was measured. An individual endocrine aggregate was defined as an area of insulin-positive tissue separated from any other adjacent insulin positive tissue by ≥50 µm of non-endocrine tissue (insulin-negative). The area of islets in the pancreas of healthy non-diabetic control C57Bl/6 mice was measured as a control. Area was determined using image J software (http://rsbweb.nih.gov/ij/) and the vascular density (number of CD34-positive ECs per square millimeter of endocrine tissue) was determined.

### Statistical analysis

Statistical analysis used Student's *t* test or ANOVA, as appropriate. Two-way repeated measurement ANOVA was used with Bonferroni's post hoc test to analyze repeated measurements in the same animal at different time points. A Kaplan–Meier survival curve was used to identify differences in the time to cure between groups. A *p* value of *p*<0.05 was considered significant. All data are expressed as means ± SEM.

## Results

### Morphology and vascularisation of pelleted and dispersed islet grafts

**Figure 1:**
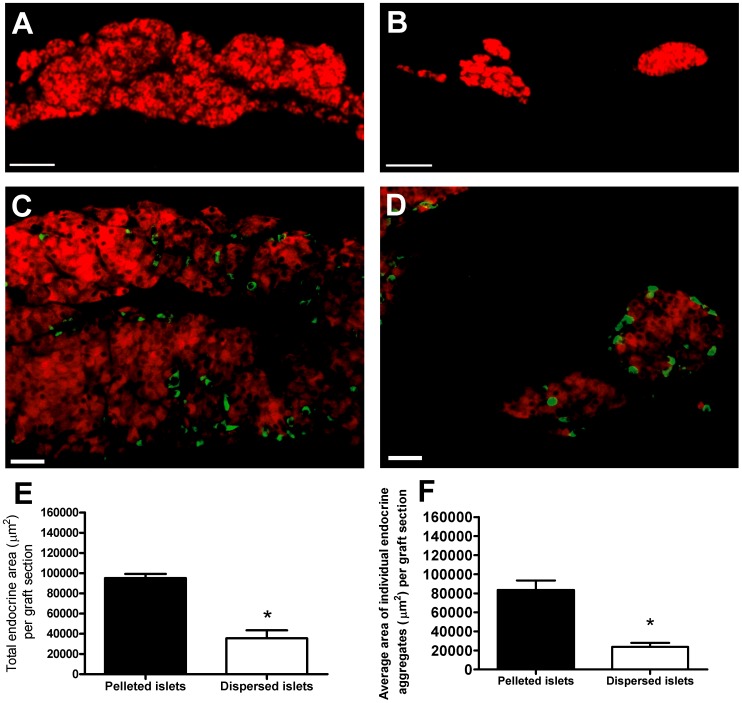
Morphology of pelleted and manually dispersed islet grafts. A, B Representative sections of pelleted islet (a) and manually dispersed islet grafts (b) at one month post transplantation beneath the kidney capsule, immunostained with insulin antibodies. A. Pelleted islet graft, where islets have typically aggregated to form a single amorphous endocrine mass in which the spherical morphology of individual islets can no longer be discerned. B. Dispersed islet graft, where large endocrine aggregates formed by the fusion of multiple islets are not present, but where multiple individual islets can still be seen in individual graft sections, original magnification ×100, scale bars are 100 µm. C, D Representative sections of pelleted islet (c) and manually dispersed islet grafts (d) at one month post transplantation, dual stained with insulin (red) and glucagon (green) antibodies, original magnification ×200, scale bars are 25 µm. E. Total endocrine area in graft sections; n = 4 animals per transplant group, *p<0.05, Student's *t* test. F. Average individual endocrine aggregate area in graft sections; n = 4 animals per transplant group, *p<0.05 vs. pelleted islet grafts, Student's *t* test.

At 1 month post transplantation graft-bearing kidneys were harvested and visualised under a dissecting microscope. In the grafts of mice transplanted with pelleted islets, individual islets could not be distinguished from each other within the single mass of compacted islets. Whereas, individual islets were clearly discernible in the dispersed islet grafts and occupied a larger area beneath the kidney capsule compared to pelleted islets. Figure 1 shows the morphology of graft material retrieved at 1 month post transplantation, demonstrating that the technical procedure of manually spreading islets beneath the kidney capsule was able to maintain the typical size and morphology of endogenous pancreatic islets, in comparison with the amorphous mass of endocrine tissue formed in the control pelleted islets transplant group. Insulin immunostaining of graft sections from mice transplanted with pelleted islets revealed a single amorphous mass of aggregated insulin-positive endocrine tissue in the majority of sections analysed (Figure 1a), resulting from the fusion of individual islets beneath the kidney capsule. In contrast, for most of the graft sections from dispersed islet transplant recipients, there was little evidence of any fusion between individual islets, with the spherical morphology of individual islets still clearly discernible (Figure 1b). Immunostaining for glucagon-positive alpha cells indicated that the core-mantle segregation of islet endocrine cells was disrupted in pelleted islet grafts (Figure 1c), whereas alpha cells were located at the periphery of individual islets in dispersed islet grafts (Figure 1d). The total endocrine area (immunostained with insulin) per graft section was reduced in dispersed islet grafts (Figure 1e), demonstrating that the isolated islets had been dispersed over a larger area beneath the kidney capsule compared to that of pelleted islet controls. The extent of islet fusion was quantified to determine the extent to which manually spreading islets at the implantation site can prevent the formation of large aggregated endocrine masses. Islet area was also quantified in endogenous pancreatic islets from healthy age-matched non-diabetic control C57Bl/6 mice as a reference to help describe the extent to which islet fusion had occurred/been prevented within grafts. The mean area of islets in the pancreas of non-diabetic mice was 19,422±1,861 µm^2^, n≥20 islets in each pancreas from 4 mice. The average area of each single endocrine aggregate per graft section in the dispersed islet grafts was approximately 25% of that seen for pelleted islet grafts (Figure 1f).

**Figure 2:**
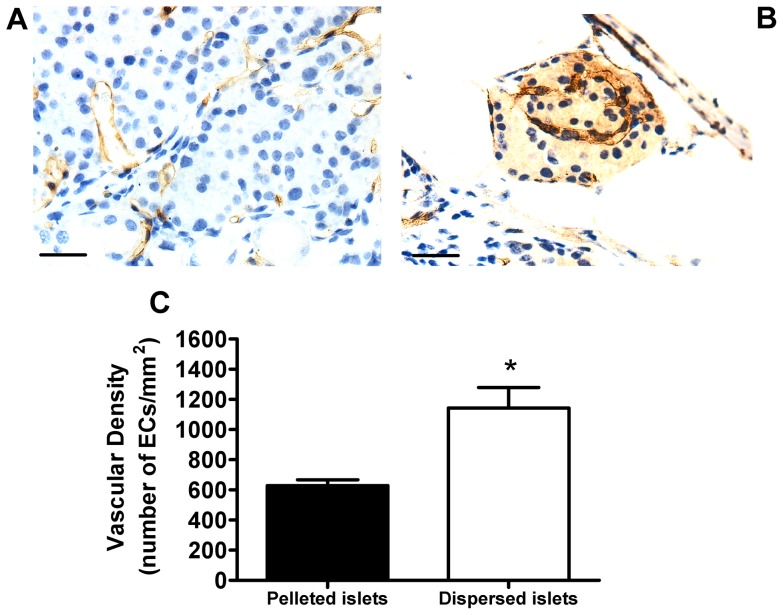
Vascular density of pelleted and manually dispersed islet grafts. Immunostaining of microvascular endothelial cells (ECs) with CD34 antibodies in pelleted (a) and dispersed (b) islet grafts. Original magnification ×400, scale bars 25 µm. C. Vascular density of endocrine components in 1 month grafts consisting of pelleted (black bar) or dispersed (white bar) islets. *p<0.05 vs. pelleted islet grafts, n = 4 animals per group, Student's *t* test.

CD34 antibodies were used to immunostain microvascular ECs in 1 month grafts consisting of pelleted and dispersed islets. The endocrine tissue of pelleted islet grafts contained large areas devoid of ECs (Figure 2a), whereas ECs were located throughout the individual islets clearly visible in dispersed islet grafts (Figure 2b). The endocrine vascular density was significantly higher in the dispersed islet grafts, compared to pelleted islet grafts (Figure 2c).

### Efficacy of pelleted and dispersed islet transplants in vivo

**Figure 3:**
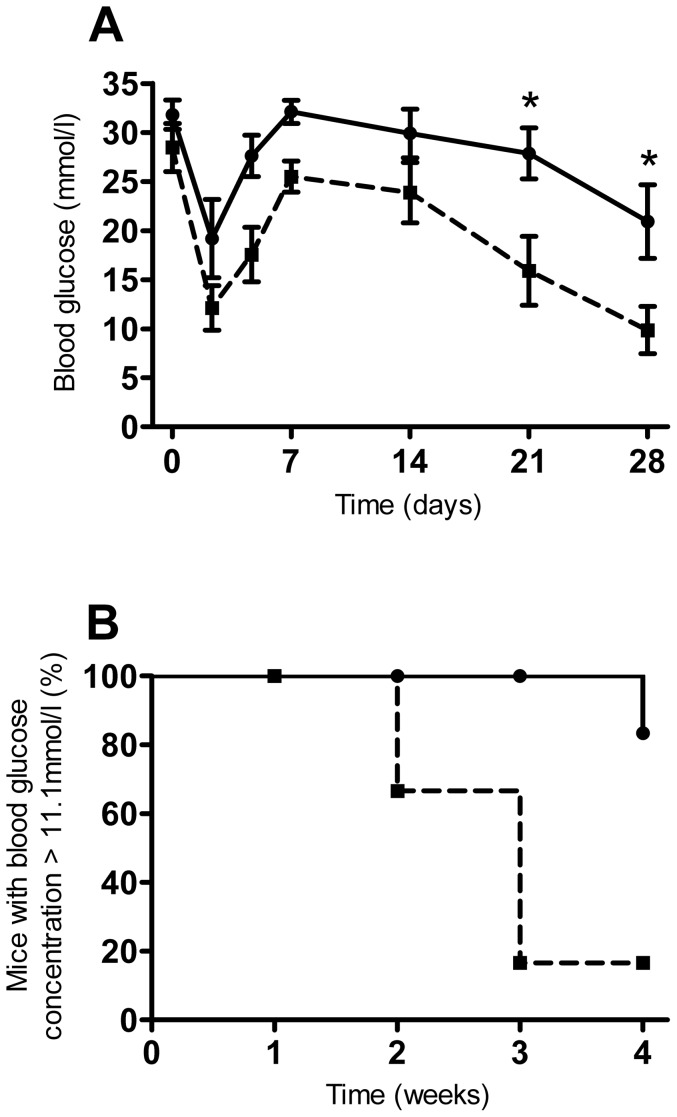
Efficacy of pelleted and manually dispersed islets in vivo. A. Blood glucose concentrations of mice with pelleted (continuous line) or manually dispersed islet transplantation (dashed line), *p<0.05, Two-Way RM ANOVA with Bonferroni post hoc test, n = 6 for both transplant groups. B. Percentage of mice remaining diabetic (blood glucose concentration >11.1 mmol/l) after transplantation as in A, *p* = 0.02 Kaplan–Meier, n = 6 for both transplant groups.

Dispersion of the islet transplant underneath the kidney capsule produced superior transplantation outcomes compared to that of islets transplanted as a single pellet/cluster of islets, as shown in Fig. 3. The average blood glucose concentrations of mice with dispersed islet transplants was significantly lower than that of mice transplanted with pelleted islets at 21 and 28 days post transplantation (Figure 3a). After one month, only 1/6 mice transplanted with pelleted islets had cured, compared to 5/6 mice with dispersed islet transplants (Figure 3b). The average time to reverse hyperglycaemia for mice with dispersed transplants was 24±2 days, with only one mouse in the pelleted islets transplant group curing at all, at 28 days post transplantation. All nephrectomised mice reverted to severe hyperglycaemia, with blood glucose concentrations of ≥33.7 mmol/l 3 days after removal of the graft-bearing kidney. In accordance, immunohistochemical analysis of the STZ-pancreata of graft recipients revealed no detectable signs of β-cell regeneration as determined by the very low levels of insulin immunoreactivity. In contrast, glucagon immunoreactivity was readily detectable in the STZ pancreata of graft recipients. One mouse in the dispersed islet transplant group was excluded from the study as histological analysis of the graft tissue clearly showed that the dispersion technique had not worked, as was evident from the large endocrine aggregates present.

### Morphology and vascularisation of matrigel-implanted islets

**Figure 4:**
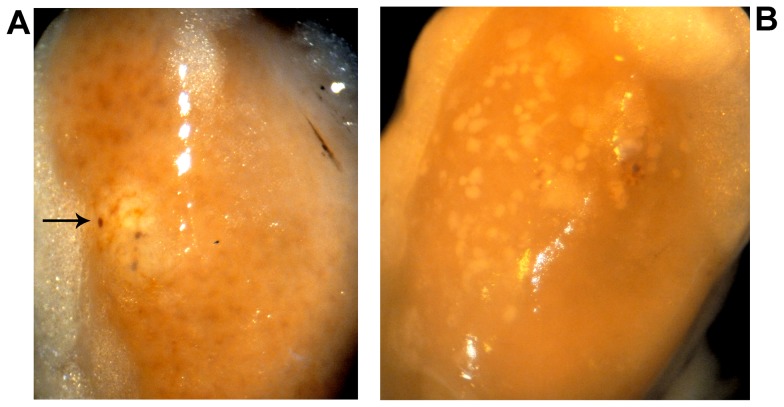
Dispersion of islets beneath the kidney capsule in matrigel plugs. Photographs of islet-graft bearing kidneys at one month post transplantation. Islets transplanted as a single islet pellet/cluster (a) and islets spread beneath the kidney capsule in matrigel (b), at one month after transplantation. Photographs were taken with a digital camera (Panasonic Lumix DMC-ZX1) through a dissecting microscope. Arrow indicates aggregated islets.

At 1 month post transplantation graft-bearing kidneys were harvested and visualised under a dissecting microscope. The implanted islets were identifiable in both transplant groups, but their appearance was clearly different, as shown in Fig. 4. The grafts of mice transplanted with pelleted islets were present as a single mass of compacted islets (Figure 4a). The grafts of mice transplanted with islets in matrigel appeared as islets dispersed over a larger area beneath the kidney capsule (Figure 4b), with little evidence of single pellets/clusters of islets in any of the mice.

**Figure 5:**
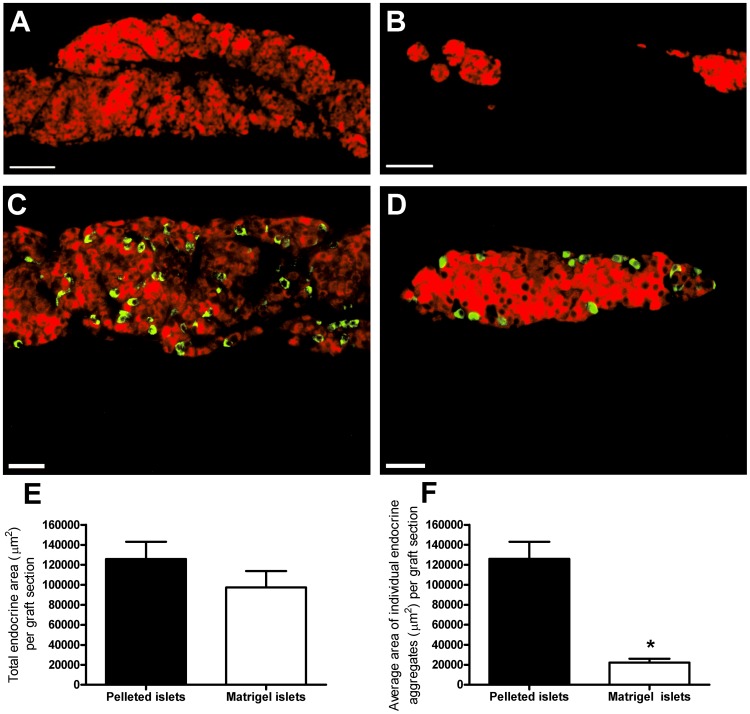
Morphology of matrigel-implanted islets. Representative sections of pelleted islet grafts (a) and matrigel-implanted islets (b) at one month post transplantation beneath the kidney capsule, immunostained with insulin antibodies, original magnification ×100, scale bars are 100 µm. A. Pelleted islet graft representing the large endocrine aggregates formed from the fusion of individual islets. B. Matrigel-implanted islet graft where the rounded morphology of individual islets separated by large areas of non-endocrine tissue, can be clearly visualised. C, D Representative sections of pelleted islet (c) and matrigel-implanted islets (d) at one month post transplantation, dual stained with insulin (red) and glucagon (green) antibodies, original magnification ×200, scale bars are 25 µm. E. Total endocrine area in graft sections; n = 4 animals per transplant group, *p*>0.2, Student's *t* test. F. Average individual endocrine aggregate area in graft sections; n = 4 animals per transplant group, *p<0.05 vs. pelleted islet grafts, Student's *t* test.

Figure 5 demonstrates the typical graft morphology of pelleted islets and islets dispersed in matrigel at 1 month post transplantation, demonstrating that dispersing islets within matrigel is able to maintain islet size and morphology more closely to endogenous pancreatic islets than that seen in pelleted islet grafts. As shown in Figure 5, insulin immunostaining of pelleted islet grafts showed a single large amorphous endocrine mass in the majority of sections analysed (Figure 5a), whereas in the islet-matrigel transplants there was very little evidence of any fusion between individual islets with the spherical outline of individual islets still clearly discernible (Figure 5b). The endocrine cell distribution was disorganised in pelleted islets grafts, with glucagon-positive alpha cells distributed throughout the aggregated endocrine mass (Figure 5c), confirming our observations demonstrated in Figure 1. Similarly to dispersed islet grafts; the normal core-mantle segregation of alpha cells was maintained in matrigel-implanted islets (Figure 5d). Although the total endocrine area (immunostained with insulin) was comparable between mice transplanted with pelleted islets and those receiving matrigel-implanted islets (Figure 5e), the graft morphology was clearly different. Quantification of islet fusion showed that the average area of each individual endocrine aggregate in the graft sections of matrigel-implanted mice was ∼15% of that seen in pelleted islet grafts (Figure 5f), indicating that this represents a reliable method for preventing islet fusion.

**Figure 6:**
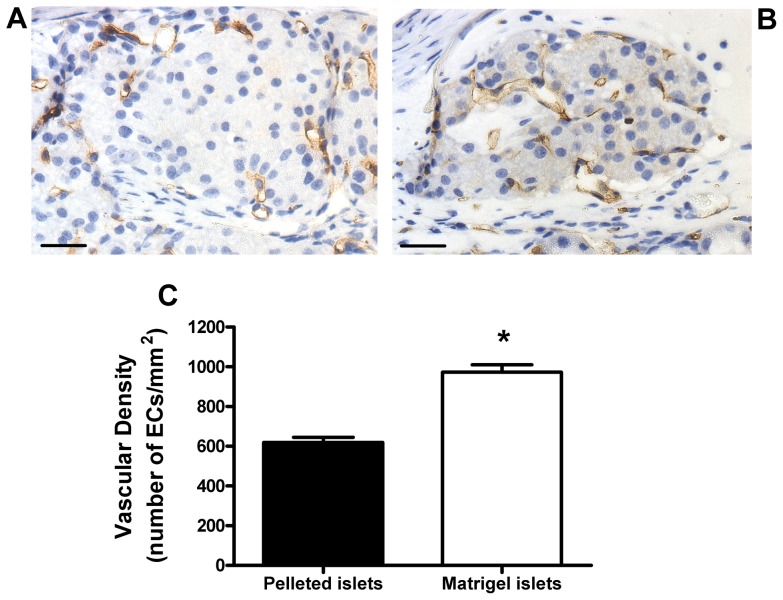
Vascular density of matrigel-implanted islets. CD34 immunostaining of microvascular endothelial cells (ECs) in pelleted islet grafts (a) and matrigel-implanted islet grafts (b) at 1 month post transplantation. Original magnification ×400, scale bars 25 µm. C. Vascular density of endocrine components in 1 month grafts consisting of pelleted (black bar) or matrigel-implanted (white bar) islets. *p<0.05 vs. pelleted islet grafts, n = 4 animals per group, Student's *t* test.

Large areas of endocrine tissue were devoid of ECs in the grafts consisting of pelleted islets (Figure 6a), whereas ECs were distributed throughout the endocrine tissue in grafts consisting of matrigel-implanted islets (Figure 6b). The vascular density of the endocrine tissue in grafts consisting of matrigel-implanted islets was significantly elevated compared to control pelleted islet grafts (Figure 6c). There were no differences between the vascularisation of matrigel implanted islets and dispersed islets (973.30±36.83 and 1,143.89±135.98 ECs/mm^2^, *p*>0.2, n = 4), nor were there any differences in the average area of individual endocrine aggregates per graft section (22,144±3,777 and 23,639±4,352 µm^2^, *p* = 0.8, n = 4). However, the total endocrine area per graft section was significantly higher in matrigel-implanted islet grafts than dispersed islet grafts (97,458±16,348 and 35,564±7,898 µm^2^, n = 4, *p*<0.05), indicating that manual dispersion of islets ensured that individual islets were spread out over a larger area beneath the kidney capsule than matrigel-implanted islets that were less spread out due to the solid matrigel support.

### Efficacy of matrigel-implanted islets in vivo

**Figure 7:**
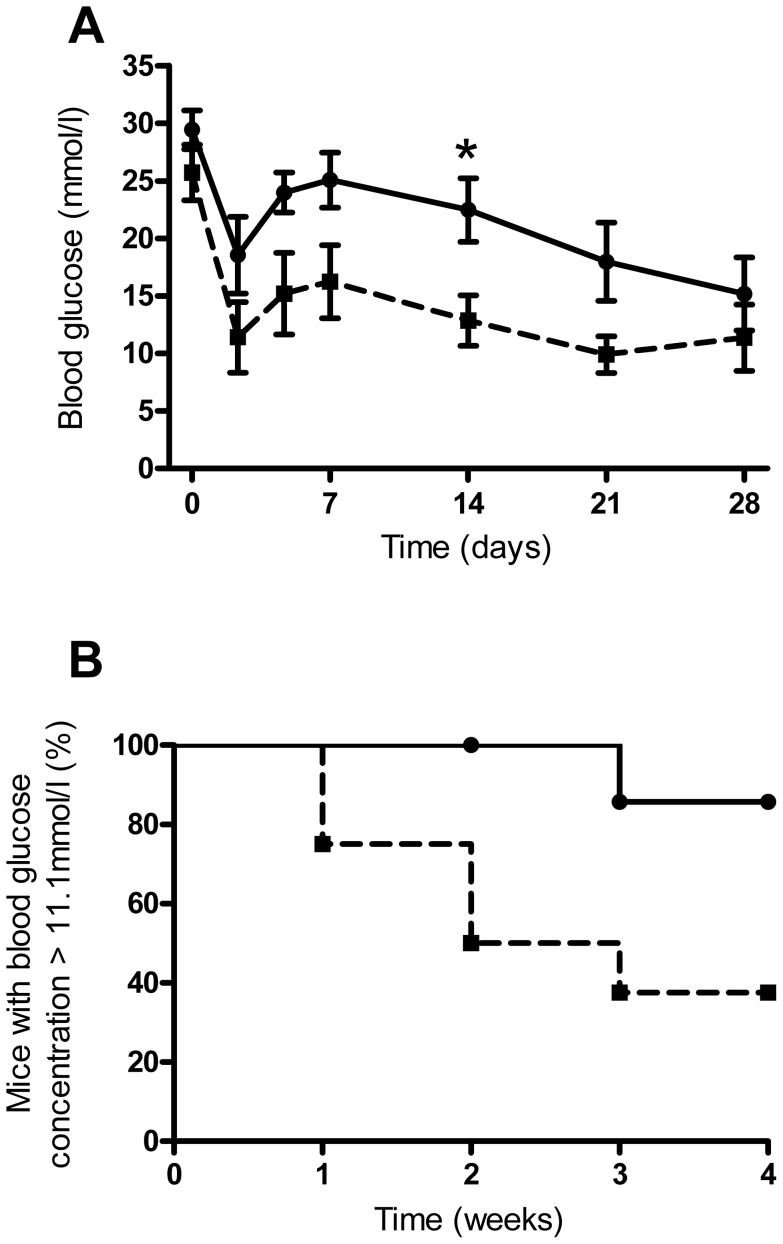
Efficacy of matrigel-implanted islets in vivo. A. Blood glucose concentrations of mice transplanted with pelleted islets alone (continuous line) or islets dispersed in matrigel (dashed line), beneath the kidney capsule, *p<0.05, Two-Way RM ANOVA with Bonferroni post hoc test, n = 7-8. B. Percentage of mice remaining diabetic (blood glucose concentration >11.1 mmol/l) after transplantation as in A, *p* = 0.02 Kaplan–Meier, n = 7-8.

Transplantation of islets in matrigel plugs produced superior transplantation outcomes to that of pelleted islets, as shown in Fig. 7. The average blood glucose concentrations of islet-matrigel implanted mice were significantly lower than in mice transplanted with pelleted islets at 14 days post transplantation (Figure 7a). The curative capacity of islets in matrigel was better than that of pelleted islets, with 5/8 islet-matrigel implanted mice curing by one month, compared to only 1/7 control mice transplanted with pelleted islets (Figure 7b). The average time to reverse hyperglycaemia for islet-matrigel implanted mice was 10±4 days, with one mouse in the pelleted islet transplant group curing after 21 days.

## Discussion

In the current study, we used a syngeneic minimal islet mass transplantation model in STZ-induced diabetic mice to demonstrate that maintaining normal islet size and morphology at the implantation site is beneficial for transplantation outcome. We used two different experimental approaches to maintain normal islet morphology by preventing islet fusion and thus limiting the formation of large amorphous endocrine aggregates. The first approach was to transplant islets at the renal subcapsular site by manually dispersing individual islets beneath the kidney capsule, as opposed to the standard procedure of transplanting them as a single pellet or cluster. In the second approach, islets were mixed with and transplanted in matrigel to ensure that the transplanted islets were physically separated beneath the kidney capsule by the solid matrigel support.

Our morphological measurements showed that manually dispersing islets beneath the renal capsule reduced the size of individual endocrine aggregates by approximately 75 percent compared to grafts of pelleted islets, consistent with maintaining normal endogenous pancreatic islet size at the subcapsular implantation site. Providing the transplanted islets with the physical support of a matrigel matrix was equally effective in maintaining individual islet morphology under the kidney capsule, producing similar effects on islet distribution and anatomy at the graft site. Immunostaining of islet α-cells for glucagon expression showed that islet architecture was maintained in both the dispersed and matrigel islet grafts, with the majority of graft sections showing a peripheral rim of α-cells surrounding the β-cell core, in contrast to the disorganised core-mantle cellular architecture in pelleted islet grafts [6]. Both methods of maintaining islet structure were also associated with significantly enhanced revascularisation of the graft endocrine tissue, as demonstrated by increased vascular density when compared to conventional pelleted islet grafts.

Immunohistochemical analysis of the STZ pancreata at one month post transplantation revealed very low numbers of insulin-positive cells and all cured mice that were nephrectomised reverted to severe hyperglycaemia (blood glucose ≥33.7 mmol/l). This is consistent with our previous observations where we demonstrated that spontaneous pancreatic β-cell regeneration is unlikely to account for improved glycaemia in high dose STZ-diabetic mice over a 1 month monitoring period. Instead, the maintenance of islet anatomy in grafts consisting of both dispersed islets and matrigel-implanted islets is associated with improved transplantation outcome in the current study. Thus, when compared to conventional islet pellets, both methods to maintain islet morphology and size enhanced the rate and frequency of reversion to normoglycaemia in STZ-induced diabetic mice and showed significant improvements in overall glycemic control. Notably, we observed an initial decrease in blood glucose in all islet graft recipients, which we believe is not physiologically relevant. Instead, this is likely to be due to extensive islet cell death [4], [5] and subsequent insulin leakage from dying cells during the immediate post transplantation period. The real differences in glycaemia are present at 2–4 weeks post transplantation when the anatomical remodelling and revascularisation process are known to be completed [17], [18].

Matrigel is a solubilised basement membrane preparation extracted from an Engelbreth-Holm-Swarm mouse sarcoma [19], in which the main components are ECM proteins such as laminin, collagen IV, fibronectin and perlecan [20]. These basement membrane proteins are involved in interactions between intraislet ECs and endocrine cells [21], [22] and a number of studies have suggested that loss of integrin signalling between islets and the surrounding ECM proteins is detrimental to islet function [21], [23], [24]. Conversely, entrapment of islets within ECM scaffolds is reported to enhance islet function [25]–[29] and survival [21], [28], [30], [31]. In the present study we did not detect any additional *in vivo* benefit of suspending the islets in matrigel over and above the improved function associated with the maintenance of islet morphology by physical dispersion below the renal capsule. This does not imply that islet-ECM interactions are unimportant, but suggests that interactions with the specific matrix components present in matrigel are neither beneficial nor detrimental for islet survival and function *in vivo* when transplanted to the renal subcapsular site. Thus, the beneficial effects of matrigel in our experimental model can be attributed to its role as a physical support to maintain islet anatomy.

There are a number of mechanisms through which maintained islet architecture may have beneficial effects on graft function and transplantation outcome in our studies. Hypoxia-related dysfunction [32] and cell death [4], [5], [33], [34] is an important confounding factor in the survival of avascular islets during the immediate post-transplantation period. Oxygen tension gradients across fused islet tissue have been demonstrated previously [35], with higher partial pressures of oxygen at the periphery of the islet graft compared with centrally located parts of the graft. Diffusion of oxygen and nutrients will be more effective in smaller endocrine aggregates enhancing their survival and function until the re-establishment of the islet vasculature. Small islet aggregates have previously been shown to be superior to large intact islets as graft material in diabetic mice, with improved transplantation outcomes being associated with reduced hypoxia-related necrosis in the small islet aggregates [36]. Importantly, this benefit of small islet aggregates over large intact islets was demonstrated using encapsulated islets which do not revascularise *in vivo*, so the improved islet function was independent of any influence on islet revascularisation.

Graft revascularisation is obviously important for subsequent function and inadequate revascularisation of transplanted islets at a number of implantation sites is associated with deleterious outcomes [37]–[40], whereas improvements in graft revascularisation are associated with improved islet function and long-term survival [41]–[43]. Our results demonstrate that maintaining individual islets at the graft site resulted in a significant enhancement of revascularisation, consistent with a previous report of superior revascularisation of small, compared to larger islets [44]. Similarly, in our previous study where we co-transplanted islets with MSCs, the resultant smaller endocrine aggregates had an enhanced vascular density compared to that of the large endocrine masses formed in mice implanted with islets alone [6].

Intra-islet interactions are known to be important for normal islet function [45], [46] and disruption of islet architecture is associated with impaired secretory responses to a range of physiological stimuli. Maintaining anatomically correct islet architecture may therefore further enhance graft function by facilitating the numerous interactions between islet cells [47] that are required for normal insulin secretion [45], [46].

Our observations using the renal subcapsular graft site are in accordance with recent studies of intramuscular islet transplantation, in which islets grafted as clusters developed central fibrosis [48], whereas transplanting the islets in a ‘pearls-on-a-string’ configuration, such that they are engrafted essentially as single islets, was associated with improved transplantation outcomes [49]. This suggests that the beneficial impact of maintaining islet anatomy during transplantation is not graft site-specific.

In conclusion, there is mounting evidence that the current intraportal route for clinical islet transplantation places the grafts into a hostile microenvironment and confers multiple and perhaps avoidable stresses upon the transplanted islets [3], so efforts are being made to identify alternative optimal implantation sites for islets. The current study suggests that preventing the fusion of islets at extrahepatic sites represents an important strategy for promoting islet engraftment, which may contribute to achieving routine single donor islet transplantation [2], [50], thereby increasing the availability of donor islet tissue and enabling the more widespread application of islet transplantation as a therapy for T1D.
